# A Pilot Study for the Neuroprotective Effect of Gongjin-dan on Transient Middle Cerebral Artery Occlusion-Induced Ischemic Rat Brain

**DOI:** 10.1155/2012/682720

**Published:** 2012-06-06

**Authors:** Yun-Young Sunwoo, Sang In Park, Yong-An Chung, Jisoo Lee, Moon-Seo Park, Kyung-Sool Jang, Lee-So Maeng, Dong-Kyu Jang, Ruth Im, Yu Jin Jung, Soon A. Park, Eun-Sun Kang, Min-Wook Kim, Young-Min Han

**Affiliations:** ^1^Institute of Catholic Integrative Medicine (ICIM), Incheon St. Mary's Hospital, The Catholic University of Korea, Incheon 403-720, Republic of Korea; ^2^Department of Radiology, Incheon St. Mary's Hospital, The Catholic University of Korea, Incheon 403-720, Republic of Korea; ^3^Chicago Medical School, Rosalind Franklin University of Medicine and Science, North Chicago, IL 60064, USA

## Abstract

In this study, we investigated whether gongjin-dan improves functional recovery and has neuroprotective effects on reducing the infarct volume after transient middle cerebral artery occlusion (MCAo). Infarct volume was measured using TTC staining and glucose utilization by F-18 FDG PET. Functional improvement was evaluated with the Rota-rod, treadmill, Garcia score test, and adhesive removal test. At 14 days after MCAo, neuronal cell survival, astrocytes expansion, and apoptosis were assessed by immunohistofluorescence staining in the peri-infarct region. Also, the expression of neurotrophic factors and inflammatory cytokines such as VEGF, BDNF, Cox-2, TNF-**α**, IL-1**β**, and IL-1**α** was measured in ischemic hemisphere regions. The gongjin-dan-treated group showed both reduced infarct volume and increased glucose utilization. Behavior tests demonstrated a significant improvement compared to the control. Also in the gongjin-dan treated group, NeuN-positive cells were increased and number of astrocytes, microglia, and apoptotic cells was significantly decreased compared with the control group in the ischemic peri-infarct area. Furthermore, the expression of VEGF and BDNF was increased and level of Cox-2, TNF-**α**, IL-1**β**, and IL-1**α** was decreased. These results suggest that gongjin-dan may improve functional outcome through the rapid restoration of metabolism and can be considered as a potential neuroprotective agent.

## 1. Introduction

Ischemic stroke is a second leading cause of death in industrialized countries and arguably the most important cause of acquired disability [1, 2]. The initial pathophysiology due to a reduction or complete blockade of regional cerebral blood flow (rCBF) results in deficient glucose and oxygen supply to the affected region [3, 4]. Ischemic stroke can be separated into three serial phases following injury: acute (minutes to hours, metabolic stress and excitotoxicity), subacute (hours to days, inflammation and apoptosis), and chronic (days to months, repair and regeneration) [5–7].

In acute stroke, the primary brain insult is largely attributed to interruption of CBF and breakdown of the blood brain barrier (BBB). The reduction of rCBF causes cerebral infarction such as middle cerebral artery occlusion (MCAo). rCBF of ischemic core was decreased 5–20% [8]. Also with rCBF recovery immediately after stroke, damaged cells in the ischemic penumbra can be rescued. Changes in rCBF and metabolism are commonly associated with brain disorders, such as traumatic brain injury and ischemic stroke [9, 10]. Also, BBB disruption after MCAo has been implicated in hemorrhagic transformation [11]. In the normal state, glucose is the predominant substrate for energy metabolism in the brain and has a tightly controlled relationship with rCBF. The glucose analogue F-18 FDG (2-[F-18]-fluoro-2-deoxy-D-glucose) is an indicator of utilization and the degree of neuronal activity in the brain [12]. Furthermore, positron emission tomography (PET) detection of F-18 FDG uptake is therefore valuable in the study of stroke, brain injury, and specific function of living animals.

In recent studies, a consideration has been given to the individual active compound alone as well as in combination with other herbs [2, 13, 14]. Numerous studies have sought novel neuroprotective substances and the beneficial effects of herbal medicines against brain ischemia have often been reported. This is related by the inhibition of inflammatory cytokines and microglia activation in brain ischemia. In traditional oriental medicine, many herbal drugs and prescriptions have in fact been used clinically for the treatment of ischemic stroke and vascular dementia. Gongjin-dan is a multiherbal formula available in Korea and China and is known as an antifatigue and antiaging agent. It has also been used as alternative medicine for the treatment of various neurodegenerative disorders. Moon et al. reported that learning and memory were enhanced following administration of gongjin-dan in the stress rat model [15]. However, experimental evidence clarifying the mechanism of gongjin-dan is limited at best.

Herein, we evaluated the effects of gongjin-dan on infarct volume and functional recovery, as well as on glucose metabolism, apoptosis and survival of living cells, and change of inflammatory cytokines and growth factors in an ischemic stroke rat model.

## 2. Methods

### 2.1. Preparation and Administration of Gongjin-dan

For this study, gongjin-dan was prepared as a mixture of *Moschus moschiferus* (175 mg), *Corni Fructus* (1.5 g), *Angelica gigantis radix* (1.5 g), and *Cervi Parvum Cornu* (1.5 g). Gongjin-dan was extracted using reflux extraction equipment in hot water for an hour and concentrated using a rotary evaporator before lyophilization. Gongjin-dan was treated with twice (40 mg/kg) per day for 14 days after the stroke.

### 2.2. Cerebral Ischemic Model

The experimental protocol used in this study was designed in compliance with the guidelines established by the Institutional Animal Care and Use Committee of Catholic University Medical School. Transient MCAo was modeled as described previously [16–18], with slight modification. Adult male Sprague-Dawley rats (270–300 g) were initially anesthetized with 5% isoflurane in 70% nitrous oxide and 30% oxygen using an induction chamber and maintained by a mixture of 2% isoflurane under temperature controlled conditions (37 ± 0.1°C) using a rectal thermometer and heating pad (Harvard Apparatus Inc., Holliston, Massachusetts, USA). The right common carotid artery (CCA), external carotid artery (ECA), and internal carotid artery (ICA) were exposed through a ventral midline incision. A 4–0 monofilament nylon suture with a rounded tip was inserted into the CCA lumen and gently advanced into the ICA until it blocked the bifurcating origin of the MCA. Cortical blood flow was measured continuously via laser doppler (Transonic system Inc., Ithaca, NY, USA) with a photodetector probe that was stereotaxically placed through a burr hole in the skull overlying frontoparietal cortex (1.3 mm posterior, 2.8 mm lateral to the bregma). Ninety minutes later, animals were reanesthetized and then reperfused by withdrawal of the suture until the tip cleared the ECA lumen. rCBF was expressed as a percentage of pre-ischemic baseline values. MCAo animals were only included in the present study if occlusion caused a decrease in rCBF to less than 30% of the original blood flow.

### 2.3. Behavior Evaluation

Following MCAo, animals were randomly divided into two experimental groups: a controls received gastric gavage with distilled water (*n* = 7). The other group received gongjin-dan (*n* = 7). Behavior was assessed with the Garcia score test, adhesive removal, Rota-rod, and treadmill test at 1, 3, 7, 10, and 14 days following MCAo by an investigator blinded to the experimental groups. Subjects were pretrained on all tests for 3 days pre-MCAo, with only exception being the Garcia test.

#### 2.3.1. Garcia Score Test

Motor behavior index was scored blind to rat groupings by the Garcia test [19, 20]. Briefly, subjects were evaluated on six criteria for a possible total score ranging from 3 to 18, with a higher score indicating better performance. Motor performance was evaluated on spontaneous activity, symmetry of movements, symmetry of forelimbs, and climbing the wall of wire cage, and sensory function was measured (while reaction to touch on and response to vibrissae touch).

#### 2.3.2. Adhesive Removal Test

For adhesive removal tests, square dots of adhesive-backed paper (113.1 mm^2^) were used as bilateral tactile stimuli on the radial wrist of both distal forelimbs [21]. The time required to remove both stimuli from each limb was recorded in five trials/day for 3 days. All animals could remove the dot within 10 sec at the end of training. So, the rats were familiarized with the testing environment. Resultant data are presented as the mean time required to removal the left dot.

#### 2.3.3. Rota-Rod Test

Subjects were pretrained on the Rota-rod motor test three times/day for 3 days pre-MCAo. The Rota-rod cylinder (Cytec Korea Inc, Seoul, Korea) was accelerated from 4 to 40 rpm within 5 minutes. Only animals capable of remaining on the Rota-rod cylinder for more than 160 seconds were included in the study and the cutoff time was 300 seconds. Data are presented as the mean duration from three trials.

#### 2.3.4. Treadmill Test

Subjects were trained on a motor-driven treadmill (Panlab, Barcelona, Spain) for 20 min/day, 3 days/week at a speed of 20 m/min prior to MCAo induction. Rats were placed on a moving belt facing away from the electrified grid forcing them to run in the direction opposite of belt movement. To avoid foot shocks (with 1.0 mA (intensity)), the rats had to move forward. Only those which learned to avoid the mild electrical shock were included in this study. The maximum velocity at which the rats could run on a motor-driven treadmill was recorded to measure 3 times [22, 23].

### 2.4. rCBF Measurements

rCBF was measured as described in previous literature [24]. Rats were anesthetized (0.8% to 1.5% isoflurane, 30% O_2_, remainder N_2_O) and rectal temperature was maintained at 37 ± 0.1°C using a feedback controlled heating system. They were then fixed to a stereotaxic apparatus with a probe placed at the level of the dura directly overlying the small burr hole used to induce MCAo. rCBF was measured at the time of MCAo onset and reperfusion. Subsequent measurements were taken during 15 min/day over the next 14 days. rCBF was expressed as a percentage of preischemic baseline values. Two investigator blinded to the experimental groups performed the rCBF recordings.

### 2.5. PET Imaging

PET imaging was performed on images taken on days 1 and 14 days after MCAo on a PET scanner using a General Electric discovery STE (Waukesha, WI, USA). Subjects were deprived of food for about 12~16 hours preceding F-18 FDG injection but had access to drinking water at all times. F-18 FDG (1.1 ± 0.04 mCi) was injected intravenously into the caudal vein, followed by a 30 min uptake period, during which the subjects remained conscious in a warm environment for optimal brain F-18 FDG uptake, by placing the entire cage on a heating pad. During brain scanning, anesthesia was maintained with katamine (80 mg/kg) and xyzine (10 mg/kg), and body temperature was kept at 37°C with a heating pad on the scanner bed. PET imaging produces 6 coronal image planes separated by 3 mm in stereotactic apparatus on pinpoint marker.

### 2.6. PET Imaging Data Analysis

PET imaging data was analyzed as per described in previous literature [10]. To assess changes in metabolism induced by artery occlusion, average regions of interest (ROI) in each hemisphere were identified in images of the coronal brain sections. The mean standard uptake value (SUV) of F-18 FDG in ROI was calculated as the averaged nCi/cc after calibration for both ipsilateral and contralateral insulated areas in the same images. The radioactivity in the contralateral area was used as a reference to normalize data obtained in the ipsilateral area, and the differential uptake ratio (DUR) was calculated [10]. The total DUR for each imaging was calculated by summation of all brain imaging. Also we confirm whether infarct volume was associated with elevated DUR for one day after MCAo (see Figure 1 in Supplementary Materials available online at doi:10.1155/2012/682720).

### 2.7. Quantitative Analysis of Infarct Volume

At 14 days after MCAo, all test subjects (*n* = 5, for each group) were deeply anesthetized with 15% urethane and sacrificed by decapitation. The brain was immediately removed and sectioned into four equally spaced (2 mm) coronal blocks using a rodent brain matrix. These sections were stained with 0.1 M PBS containing 2% 2-3-5-triphenylterazolium (TTC; Sigma, St. Louis, MO, USA) for 30 min at 37°C. The unstained area was considered to be the infract area. The injured area of brain slices was quantified using Meta-Morph imaging software (Molecular Devices Inc, Downingtown, PA, USA). The total infarct volume for each slice was calculated by summation of all brain slices.

### 2.8. Immunohistofluorescence Staining

At 14 days after MCAO, test subjects were sacrificed for histological examination. Rats were deeply anesthetized with 15% urethane and then perfused transcardially with 0.01 M PBS (pH 7.4), followed by 4% paraformaldehyde in 0.01 M PBS. The brains were removed and post-fixed for 5 h. The postfixed tissue was equilibrated with 30% sucrose in 0.1 M phosphate buffer (pH 7.2) at 4°C. The brain tissues were embedded, snap-frozen in liquid nitrogen, and stored at −70°C until use. The tissue was cut into 4 *μ*m thick coronal sections between +0.1 and 0.86 mm of the bregma from each rat for a total of three blocks. The sections were warmed up for 20 min and washed with 0.01 M PBS. Sections were blocked in normal goat serum for 1 h at room temperature. The sections were incubated at 4°C overnight with the following antibodies: mouse antiglial fibrillary acidic protein (GFAP; Millipore, Billerica, MA, USA), mouse anti-neuronal nuclei antigen (NeuN; Millipore), and mouse anti-OX-42 (Millipore). After washing, sections were incubated in Alexa 488-conjugated goat anti-mouse IgG (Molecular probe, Eugene, Oregon, USA) for 1 h at room temperature. After washing, the sections were counterstained with 4′,6-diamidino-2-phenylindole (DAPI; Sigma-Aldrich). Apoptosis was detected by the terminal deoxynucleotidyl-transferase mediated d-UTP-biotin nick end (TUNEL) assay using the In Situ Cell Death Detection Kit (Roche, Mannheim, Germany) developed using the Cy3-conjugated streptavidin (Jackson ImmunoResearch Laboratories, Bar Harbor, MA, USA). After washing, the sections were counterstained with DAPI, and then observed under both a fluorescence microscope equipped with a spot digital camera and a confocal scanning laser microscope (LSM 510, Zeiss, Oberko, Germany). Using Meta-Morph imaging program (Molecular Devices Inc), ischemic penumbra was determined by optical density of GFAP and OX-42 and counting of NeuN and TUNEL-positive cells.

### 2.9. Enzyme-Linked Immunosorbent Analysis

14 days following MCAO, rat brains were removed, and coronal sections (200 mg) from −1.0 to 1.0 mm to bregma in the ischemic hemisphere were dissected on ice and were stored at −70°C until use. Subsequently, each tissue sample was suspended in an equal weight of homogenate buffer and homogenized. The homogenate was centrifuged (10,000 g) for 30 min at 4°C. The supernatant was collected and examined using the enzyme-linked immunosorbent assay (ELISA) to detect the protein levels of neurotrophic factors and inflammatory cytokines in the ischemic rat brain. The supernatant was further analyzed to quantify the concentration of brain-derived neurotrophic factor (BDNF; Abnova, Jhongli, Taiwan), vascular endothelial growth factor (VEGF; R&D system, Minneapolis, USA), cycloxygenase-2 (Cox-2; Immuno-Biological Laboratories, Gunma, Japan), tumor necrosis factor-*α* (TNF-*α*; R&D system), interleukin-1*α* (IL-1*α*; R&D system), and interleukin-1*β* (IL-1*β*; R&D system) in strict accordance with the manufacturer's protocols.

### 2.10. Statistical Analysis

The behavior tests, cerebral ischemic volume, and cell count of apoptotic cells for both rat groups were subjected to one-way ANOVA with post hoc analysis, independent *T*-test, or Mann-Whitney *U* test. Data are presented as the mean value ± standard deviation of mean. Probability values less than 0.05 were considered statistically significant.

## 3. Results

### 3.1. Behavior Test

**Figure 1:**
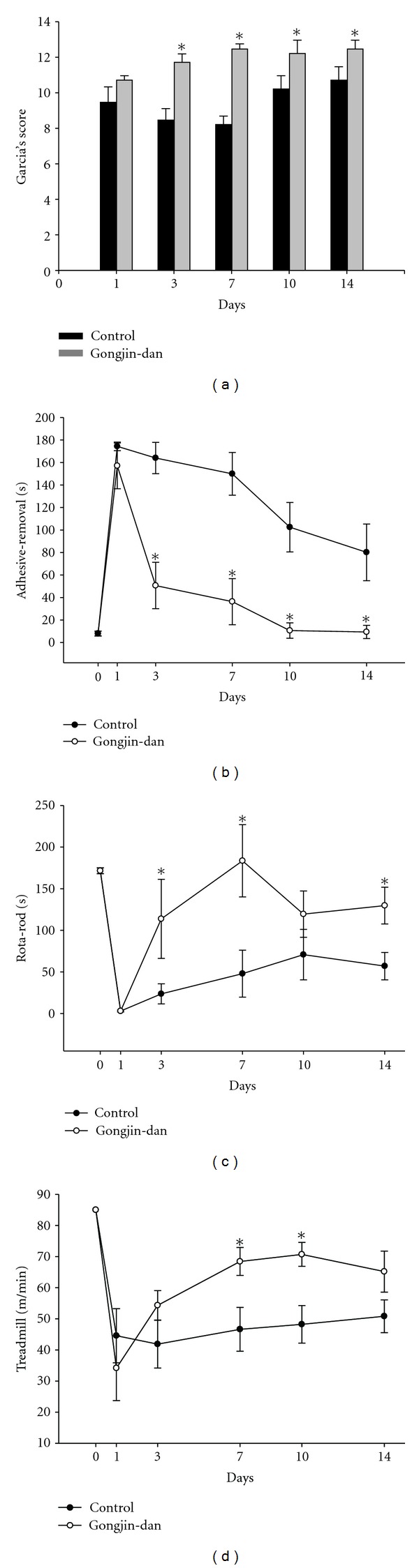
Effect of gongjin-dan on functional recovery after MCAo. Behavior performances in the Garcia's score (a), adhesive removal (b), Rota-rod (c), and treadmill test (d) from 1 to 14 days after MCAo. Behavior tests were significantly different at each time point starting at 3 days after MCAo. Data are expressed as mean ± SD, *P < 0.05.

In this study, behavior tests were performed both prior to MCAo as well as 1, 3, 7, 10, and 14 days after MCAo. We observed an overall mortality 25% in this model. The animals died between 3 days and 7 days after MCAo model. Garcia score, adhesive removal, Rota-rod, and treadmill tests were done (Figure 1). Garcia score was 18 in the normal group. At 3 days after MCAo, scores were significantly higher in the gongjin-dan group compared to the control group (11.75 ± 0.5 versus 8.5 ± 0.6, *P* < 0.05). Similarly, significant differences between the two groups were also noted in the 14 days (12.5 ± 0.5 versus 10.75 ± 0.7, *P* < 0.05). Performance on the adhesive removal test was also compared between both groups. Gongjin-dan-treated group are significantly different 3 days after MCAo compared with control group (50.6 ± 20.5 versus 163.9 ± 13.9, *P* < 0.05) (Figure 1(b)). Although no difference in Rota-rod assessment was noted on day 1 after MCAo, significant behavior recovery was demonstrated in the gongjin-dan-treated group at 3 days after MCAo (23.7 ± 12 versus 113.75 ± 47, *P* < 0.05) (Figure 1(c)). The maximum speed at which the rats could run on a motor driven treadmill was recorded. Three days after MCAo, the maximum velocity on the treadmill test indicated its maximum deficit. There was no significant difference among the groups. At 7 days after MCAo, the gongjin-dan treated group showed a greater functional recovery than the control (68.4 ± 4.5 versus 46.6 ± 7, *P* < 0.05) (Figure 1(d)). Maximum velocity identified the greatest velocity at 10 days after MCAo. At 14 days after MCAo, maximum velocity was low compared with velocity at 10 days. This may be due to the weight variation between different experiments (data not shown). These results suggest that gongjin-dan can promote the restoration of motor function in rat stroke model.

### 3.2. Infarct Volume and PET-CT

**Figure 2:**
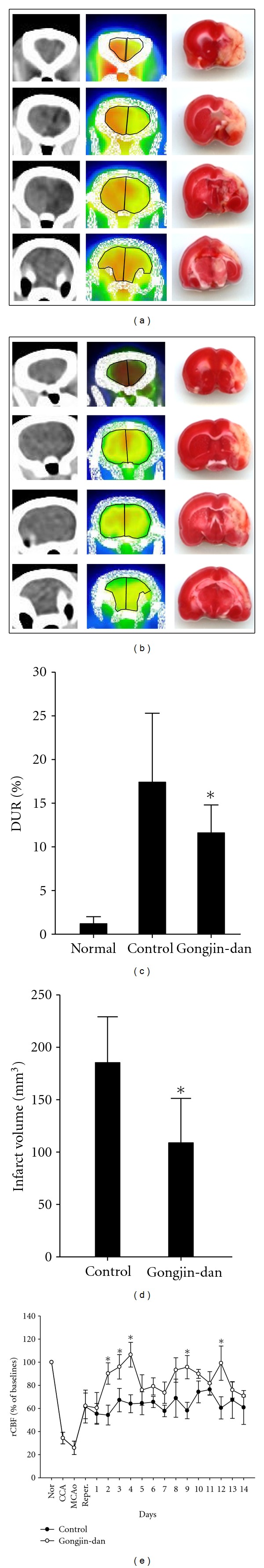
Effect of gongjin-dan on infarct volume, glucose metabolism, and regional cerebral blood flow (rCBF) after MCAo. Images shown in the PET images and TTC-stained section of control (a) and gongjin-dan treatment group (b) at 14 days. ROI by F-18 FDG uptake was revealed as differential uptake rations (DURs) (c). Infarct volume was then analyzed using Meta-Morph program (d). In gongjin-dan treatment group, infarct volume was decreased compared to the control group. Also glucose uptake was increased significantly compared with control group. rCBF was significantly increased compared with control group (e). These effects were observed for 3, 4, 5, 8, 9, and 12 days after MCAo. Data are expressed as mean ± SD, *P < 0.05.

The neuroprotective effect of gongjin-dan was evaluated by measuring the infarct volume at 14 days after MCAo. The brain of rats was stained with TTC to obtain the infarct volume and was calculated by measuring the area of infarct.  Figure 2 is a photograph typical of TTC-stained sections of treatment and control groups. As seen in Figure 2, infarct volume of the gongjin-dan group was significantly decreased compared to the control (108.8 ± 42 versus 185 ± 43 mm^3^, *P* < 0.05) (Figure 2(d)). PET-CT has long been demonstrated as a marker of glucose metabolism in ischemia and infarction [25, 26]. In our studies, PET imaging data was interpreted in terms of DUR. With the contralateral side as baseline, the metabolic change of transient MCAo in an ipsilateral area was calculated by ROI value. As seen in Figure 2(a), the quantification of the ROI radioactivity was decreased in glucose uptake following MCAo. From these control data, a DUR in the ischemic region was significantly increased compared with normal. A DUR of gongjin-dan treatment was decreased compared to the control (11.6 ± 3.2 versus 17.4 ± 7.9%, *P* < 0.05). This data suggests that protected ischemic area by gongjin-dan could uptake glucose metabolism.

### 3.3. Restoration of rCBF

To investigate whether gongjin-dan treatment improved rCBF, the rCBF was measured by laser doppler. Baseline rCBF recorded just prior to MCAo surgery was applied to express values as a percentage. Following reperfusion, rCBF was maintained at 60%. 2 days after MCAo, the gongjin-dan treatment group improved to near-normal rCBF level. In contrast, rCBF value in the control group for 14 days was similar to the rCBF value of reperfusion. This suggests that gongjin-dan could potentially halt the pathological cascade of stroke. Also, it is likely that gongjin-dan promotes behavior recovery via rCBF and glucose metabolism restoration, thereafter blocking neurosis.

### 3.4. Activating Astrocyte, Microglia, and Neuronal Death

**Figure 3:**
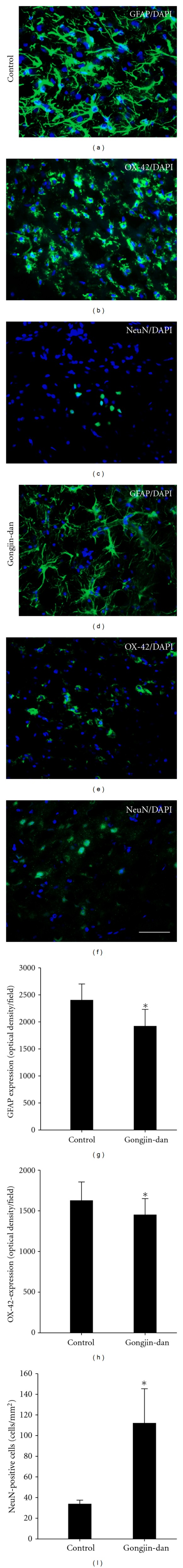
Quantitative analysis of immunoreactivities of GFAP, NeuN, and OX-42 at days on peri-infarct area after MCAo. Histological analysis was shown into GFAP, NeuN, OX-42 staining at 14 days after MCAo. Quantitative immunoreactivities for groups are shown as bar graphs on the right side of each panel. The expression of GFAP in gongjin-dan group was significantly decreased compared with control group. NeuN-positive cells also showed difference in the control group. There were many NeuN-positive cells in gongjin-dan group compared to control group. The expression of OX-42 in gongjin-dan group was significantly decreased compared with control group. In the gongjin-dan, the number of activated microglial cells was decreased and the number of neuronal cells was increased in the peri-infarct area. Data are expressed as mean ± SD, *P < 0.05.

We observed the number of astrocytes, microglia, and neurons in the peri-infarct regions at 14 days after MCAo. In gongjin-dan group, GFAP-positive cells and OX-42-positive cells showed significant decrease in the optical density compared to the control group (Figures 3(g) and 3(h)). Also NeuN-positive cells showed higher compared to the control group (Figure 3(i)). These results suggest that the inhibitory effects of GFAP and microglial activation may contribute to its neuroprotective effects in MCAo, thereby enabling a number of NeuN-positive cells to survive in peri-infarct region.

### 3.5. Apoptotic Cell Death

**Figure 4:**
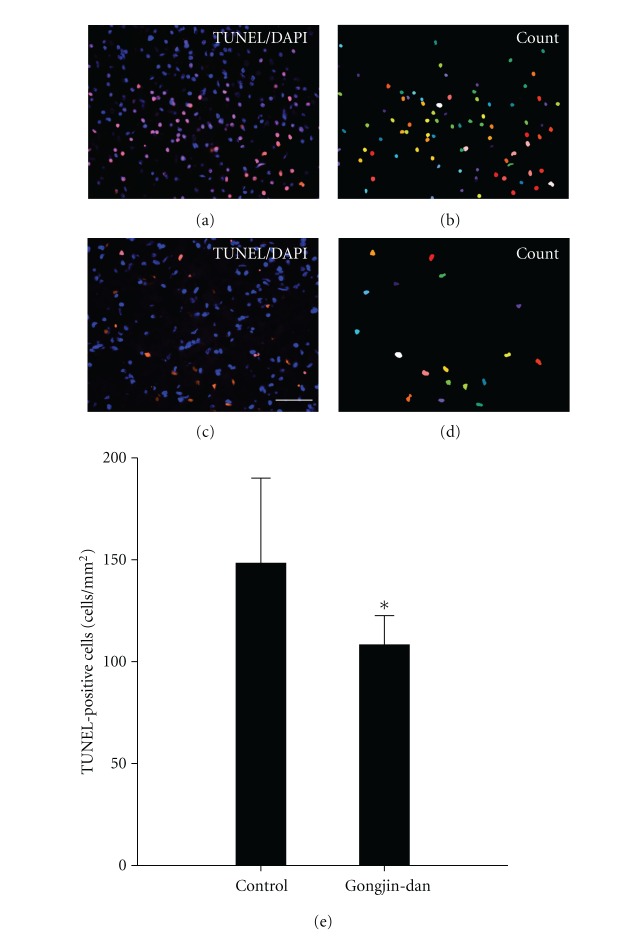
Effect of gongjin-dan on the number of apoptotic cells after MCAo. TUNEL staining was used to identify apoptotic cells. (a) Apoptotic cells were identified by immunofluorescence Cy3 (red). TUNEL positive cells were counted using Meta-Morph program (b, d). TUNEL positive cells show the quantitative analysis data (e). The number of TUNEL positive cells was significantly decreased in the drug treated group compared to the control group. Data are expressed as mean ± SD, *P < 0.05, Scale bars denote, 50 *μ*m.

We observed the number of apoptotic cells in the peri-infarct regions at 14 days after MCAo model. Apoptotic cells were recognized by the identification of DNA fragmentation using the TUNEL method. Gongjin-dan-treated groups had reduced TUNEL-positive cells compared to the control group (108 ± 14 versus 148 ± 41, *P* < 0.05) (Figure 4(e)). These results suggest that the gongjin-dan-treated group effectively prevents expansion apoptotic cell death in MCAo model.

### 3.6. Quantitative Analysis of Neurotrophic Factors and Inflammatory Cytokines

**Figure 5:**
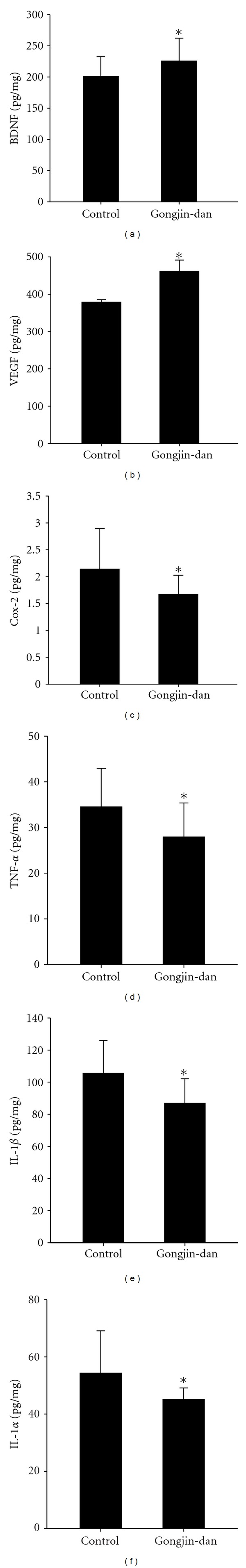
Expression of growth factors and inflammatory cytokines in the ischemic rat brain. BDNF, VEGF, Cox-2, TNF-*α*, IL-1*β*, and IL-1*α* were detected by ELISA at 14 days after MCAo. Protein levels of these factors show the quantitative analysis data (a–f). The expression of BDNF and VEGF was significantly increased compared with the control group at 14 days after MCAo (a, b). Also, inflammatory cytokines such as Cox-2, TNF-*α*, IL-1*β*, and IL-1*α* were significantly decreased compared with the control group (c, d, e, f). Data are expressed as mean ± SD, *P < 0.05.

We hypothesized that gongjin-dan may protect an injured area following a stroke by elevating neurotrophic factors and by reducing inflammatory cytokines. We used the ELISA to detect protein levels of neurotrophic factors and inflammatory cytokines in the ischemic rat brain. At 14 days after MCAo, expression of BDNF and VEGF was significantly high compared to the control group (Figures 5(a) and 5(b)). Also inflammatory cytokines such as Cox-2, IL-1*α*, IL-1*β*, and TNF-*α* were reduced as compared with control group (Figures 5(c), 5(d), 5(e), and 5(f)). These data suggest that an increment of neurotrophic factors and a decrease of inflammatory cytokines by gongjin-dan could protect cerebral infarction and promote behavior function recovery.

## 4. Discussion

During stroke, CBF is reduced and causes a decrease of oxygen supply to the brain and an increase of glutamate, inflammatory mediators, and free radicals. As a result, cellular necrosis and delayed cell death occur. In this study, we examined the effect of the gongjin-dan treatment on transient MCAo model. We investigated (1) whether the infarct volume decreases, (2) whether blood circulation and glucose metabolism improve (laser Doppler and PET-CT were performed for measurement), (3) whether there is functional recovery, and (4) whether neuronal cells in peri-infarct region can be protected from death.

 We performed TTC staining in MCAo model to identify the infarct volume. The gongjin-dan-treated group showed a more diminished infarct volume as compared with the control group. Additional immunostaining data demonstrated that gongjin-dan suppressed the increase of GFAP- and OX-42-positive cells in the peripheral area of ischemic infarct. This suggested that gongjin-dan may protect against the infarct expansion through secondary injury caused by activated astrocytes and microglia. Also, DNA breakage was investigated by TUNEL staining, which can identify apoptosis and necrosis. The gongjin-dan-treated group demonstrated more reduced TUNEL-positive cells as compared with control group. NeuN-positive cells were increased compared to control group as well. These results suggest that gongjin-dan acts as a neuroprotector by effectively preventing the expansion of astrocytes, microglia and apoptotic neuronal death in rat MCAo model.

Also, previous studies have reported that sudden destruction of BBB can reduce CBF level considerably in injured brain region. In our study, after measuring CBF for 14 days, the result was suggestive of the possibility that gongjin-dan can improve blood circulation by restoring rCBF. To support our hypothesis, we measured brain metabolic activity by using F-18 FDG PET-CT. Previous reports have shown that the increased uptake of F-18 FDG is related to the functional restoration [10, 27]. In the normal rat, there was no difference between the ROI values of the bilateral sides but the value of the injured region was quite reduced. However, in our study, the gongjin-dan-treated group demonstrated considerable reduction of DUR compared to the control group 14 days after MCAo. These results suggest that gongjin-dan may improve functional recovery by increasing rCBF, oxygen supply and glucose utilization of the brain.

After stroke, rats demonstrate disruption of sensorimotor ability and cognitive function. A lot of earlier studies have measured behavioral function by Garcia score [19], adhesive removal [21], and Rota-rod, treadmill test [23]. These behavioral tests in the gongjin-dan-treated group showed significant functional improvement in comprehensive motor and sensory test from 3 days to 14 days after MCAo. Our data suggests that gongjin-dan can promote the restoration of motor function in stroke model at an early stage. Moon et al. reported that gongjin-dan improved memory and learning [15]. They insisted that gongjin-dan promotes secretion of nerve growth factor resulting in blocking of apoptotic cell death, leading to improved neuronal survival. Neurotrophic factors such as NGF and BDNF have been thought as binding Trks receptor [28]. The activation of these pathways regulates neuronal survival, differentiation, and synaptic transmission [29]. Also, it protects neuron in various circumstances such as hypoglycemia [30] and ethanol neurotoxicity [31]. We suggest that gongjin-dan can induce activation of neurotrophic factors. Also among the components of gongjin-dan are included the phosphatidylcholine and choline [32]. Phosphatidylcholine has been reported to have an antioxidant effect, improve memory, and help nourishing brain [33, 34]. Additionally, it has been shown to improve cholesterol profile and blood circulation. Choline, a known precursor of acetylcholine, carries an important role in cell membrane function and signal transmission between neurons. It is an important neurotransmitter related to storing memory [35, 36]. These components are also known to improve neurological deficits and have neuroprotective effect on various central nervous system injury models including transient cerebral ischemia. Although our data is limited for detailed explanation of the mechanism of action of gongjin-dan, these mechanisms can have neuroprotective effect of the drug. VEGF promotes angiogenesis and neurogenesis into ischemic penumbra, where it is increased to supply blood in the brain [37]. In our study, the gongjin-dan-treated group demonstrated considerable expression of BDNF and VEGF compared to the control group 14 days after MCAo. Furthermore, TNF-*α*, IL-1*β*, IL-1*α*, and Cox-2 are well-studied cytokines related to inflammatory responses in stroke, and both appear to exacerbate ischemic damage [38–40]. Most of these factors were produced by microglia in the ischemic brain and act as death ligands and initiate the death-receptor signal pathway. Inhibition of these factors has been shown to be beneficial in neurological outcome and infarct volume in stroke. In our study, there is a possibility that the reduction in inflammatory cytokines by gongjin-dan may have neuroprotective effects in MCAo.

In conclusion, the result of our study suggests that gongjin-dan promotes secretion of neurotrophic factors such as BDNF and VEGF and inhibits inflammatory cytokine such as TNF-*α*, IL-1*β*, IL-1*α*, and Cox-2, thereby exerting its neuroprotective effect in rat MCAo model. Also, functional recovery can be achieved as a result of increased glucose metabolism through improved rCBF. Further studies are needed to identify the dose-response effect and the precise mechanism of action of this novel drug.

## Supplementary Material

PET Imaging Data Analysis and Quantitative Analysis of Infarct Volume. We confirm whether infarct volume was associated with brain metabolism for one day post-MCAo. At 1 day after MCAo model, rat (n=5) was injected intravenously F-18 FDG into the caudal vein. PET imaging produces 6 coronal image planes separated by 3 mm in stereotatix apparatus on pinpoint marker. After sacrifice at scanned rats, the brain was removed and sectioned into four equally spaced (2 mm) coronal blocks using a rodent brain matrix. These sections were stained 2% TTC. The injured area of brain slices were quantified using Meta-Morph program. The total infarct volume for each slice was calculated by summation of all brain slices. To assess changes in metabolism induced by MCAo, average ROI in each hemisphere were identified in images of the coronal brain sections. The mean SUV of F-18 FDG in ROI was calculated as the averaged nCi/cc after calibration for both ipsilateral and contralateral insulated areas in the same images. The radioacitivity in the contralateral area was used as a reference to normalize data obtained in the ipslateral area, and the DUR was calculated. The total DUR for each imaging was calculated by summation of all brain imaging.
