# Supplementary material for: A Pilot Study for the Neuroprotective Effect of Gongjin-dan on Transient Middle Cerebral Artery Occlusion-Induced Ischemic Rat Brain
Source: Evid Based Complement Alternat Med. 2012 Jun 6;2012:682720. doi: 10.1155/2012/682720 (PMC3375177; doi:10.1155/2012/682720)
Supplement: Supplementary file 1 — PET Imaging Data Analysis and Quantitative Analysis of Infarct Volume. We confirm whether infarct volume was associated with brain metabolism for one day post-MCAo. At 1 day after MCAo model, rat (n=5) was injected intravenously F-18 FDG into the caudal vein. PET imaging produces 6 coronal image planes separated by 3 mm in stereotatix apparatus on pinpoint marker. After sacrifice at scanned rats, the brain was removed and sectioned into four equally spaced (2 mm) coronal blocks using a rodent brain matrix. These sections were stained 2% TTC. The injured area of brain slices were quantified using Meta-Morph program. The total infarct volume for each slice was calculated by summation of all brain slices. To assess changes in metabolism induced by MCAo, average ROI in each hemisphere were identified in images of the coronal brain sections. The mean SUV of F-18 FDG in ROI was calculated as the averaged nCi/cc after calibration for both ipsilateral and contralateral insulated areas in the same images. The radioacitivity in the contralateral area was used as a reference to normalize data obtained in the ipslateral area, and the DUR was calculated. The total DUR for each imaging was calculated by summation of all brain imaging. [file 682720.f1.doc]

**Supplementary Figure 1.** Image of PET and TTC staining one day after MCAo. Uptake of F-18 FDG induced by MCAo was reveal in the PET image and infarct areas were identified by 2% 2-3-5-triphenylterazolium (TTC) staining at one day post-MCAo (A, B). ROIs by F-18 FDG uptake were revealed differential uptake rations (DUR) (C). Infarct volume was then analyzed using Meta-Morph program (D). Infarct volume was associated with elevated DUR. Data are expressed ad mean ± SD, **P* < 0.05


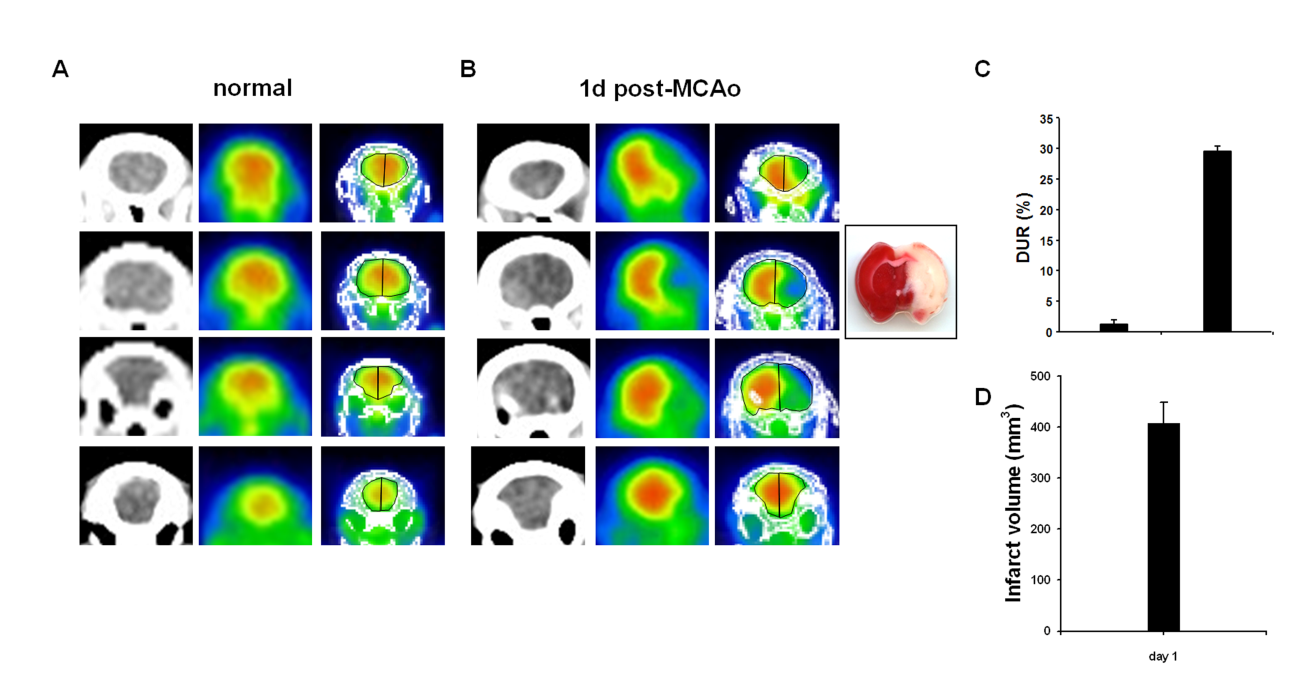
**Supplementary Figure 1**
